# High-Performance SAW Low Temperature Sensors with Double Electrode Transducers Based on 128° YX LiNbO_3_

**DOI:** 10.3390/mi13111912

**Published:** 2022-11-04

**Authors:** Jiajun Zhu, Hongliang Wang, Feng Zhang, Qi Ding

**Affiliations:** Key Laboratory of Instrumentation Science & Dynamic Measurement, Ministry of Education, North University of China, Taiyuan 030051, China

**Keywords:** surface acoustic wave (SAW), 128° YX LiNbO_3_, low temperature sensor, high Q value, structure parameters, double electrode transducers

## Abstract

Low temperature measurement is crucial in deep space exploration. Surface acoustic wave (SAW) sensors can measure temperature wirelessly, making them ideal in extreme situations when wired sensors are not applicable. In this study, 128° YX LiNbO_3_ was first introduced into low temperature measurements for its little creep or hysteresis in cryogenic environments and affordable price. The finite element method was utilized to raise the design efficiency and optimize the performance of SAW sensors by comparing the performance with different interdigital transducer (IDT) structure parameters, including the height of electrodes, pairs of IDTs, reflecting grid logarithm and acoustic aperture. Once the parameters were changed, a novel design of high-performance SAW temperature sensors based on 128° YX LiNbO_3_ with double electrode transducers was obtained, of which the Q value could reach up to 5757.18, 4.2-times higher than originally reported. Low temperature tests were conducted, and the frequency responsiveness of SAW sensors was almost linear from −100 °C to 150 °C, which is in good agreement with the simulation results. All results demonstrate that double electrode transducers are considerably efficient for performance enhancement, especially for high-Q SAW sensors, and indicate that LiNbO_3_ substrate can be a potential high-performance substitute for cryogenic temperature measurements.

## 1. Introduction

Cryogenic temperature measurement is crucial in a variety of applications, including Earth polar expeditions, deep space exploration and biological experiments [1]. So far, electrical sensors such as resistance temperature devices and thermocouples have been widely used in cryogenic temperature measurements [2]. However, a big risk exists when electrical wires are introduced to experiments with superconducting lines and magnets, making electrical sensors inadequate for applications. In addition, they are prone to premature failures in chemical and biological environments [3]. Optical wires are also a substitute for cryogenic measurement. However, the use of optical wires to implement cryogenic temperature sensors adds additional vacuum-based packaging constraints on the cryogenic system, including unwanted radiation effects from the wired parts [4].

In recent years, great progress has been made in utilizing surface acoustic wave (SAW) technology since the Rayleigh wave was first proposed by Lord Rayleigh in 1885 [5]. SAW-based temperature sensors are attractive options for the accurate measurement of temperature in harsh environments on account of their full compatibility with wireless data transmission and battery-loss operation [6]. A great deal of research has been dedicated to SAW high temperature sensors to date. In 2016, Aubert et al. prepared Ir-based composite electrodes in their SAW temperature sensor, which can measure up to 800 °C stably. Soon afterward, their team used Ta as the adhesive layer in the electrode to prepare a SAW temperature sensor capable of stable operation at 1000 °C for at least 30 min [7,8]. 

However, only a few studies exist on cryogenic temperature sensors based on SAW technology. In a very small number of studies, Alexandru Müller and his team have made great contributions. In 2015, they proposed the method of utilizing the Sezawa guided propagation mode based on GaN/Si to measure as low as −260 °C with the temperature coefficient of frequency (TCF) within 70–74 ppm/°C [9]. Subsequently, in 2021, they manufactured one-port SAW resonator structures on thin AlN layers deposited on Si and glass substrates, which can measure in the −267 °C–150 °C temperature range, and the TCF can be improved to 117 ppm/°C simultaneously [10]. Undoubtedly, Alexandru Müller and his team have provided useful guidance for the cryogenic temperature measurement utilizing SAW technology. However, the film-grown technology of GaN and AlN is under-developing, making it difficult to obtain liable and even GaN and AlN film. Meanwhile, GaN and AlN film is expensive, making it not conductive for massive popularity in SAW cryogenic temperature sensors. In 2019, Giovanni Gugliandolo et al. characterized SAW devices produced by Murata down to 20 K to evaluate the use as temperature sensors. However, the structure and the materials of the SAW resonators were not reported, and the sensitivity needed to be improved [11]. SAW cryogenic temperature sensors based on Langasite (LGS) substrates were also studied by Xu et al. for its large second-order temperature coefficient of frequency. A larger temperature coefficient of frequency of −167 ppm/K was then reported in the range of −60 °C–700 °C [12]. Similarly, the LGS substrates are also too expensive to apply on a large scale in cryogenic temperature measurement. To address this problem, a new high-performance piezoelectric material, which can not only cover the range from cryogenic temperature to room temperature, but also cost reasonably, is urgently needed. 

Lithium niobate (LiNbO_3_), as one of the most commonly used piezoelectric materials, has a negative temperature coefficient of frequency (TCF), which means its resonant frequency decreases with temperature increment [13]. Meanwhile, it has trigonal crystal symmetry structure (3 m point group). Among this, 128° YX-cut LiNbO_3_ substrates have a high electromechanical coupling coefficient, high velocity and pure Rayleigh wave mode, making them suitable for fabricating SAW temperature sensors. To date, no SAW low temperature sensors based on LiNbO_3_ have been reported. However, some research has indicated that LiNbO_3_ can be a potential material for cryogenic applications. In 2019, creep and hysteresis were directly measured by John Beamish et al. at temperatures between 0.1 K and 310 K for shear displacements of LiNbO_3_ [14]. As a result, no creep or hysteresis was seen for the single domain lithium niobate transducer, which was attractive for precise measurement applications that involved cryogenic temperatures.

In this study, high-performance SAW low temperature sensors with double electrode transducers based on 128° YX LiNbO_3_ were designed and fabricated to enhance the performance, especially the Q value, and verify its performance in low temperature environments. The finite element method (FEM) simulation was utilized to simulate the structure changes to optimize the performance of the sensors. Types of transducers were also studied to analyze the influence on performance. Double electrode transducers were first introduced into SAW temperature sensors to enhance the Q value and reduce the insertion loss. An RF network analyzer and high-low temperature chamber were used to test the performance of the manufactured SAW low temperature sensors and discover the relationship between temperature variation and resonant frequency shift. A higher Q value of 5757.18 was obtained. In addition, in the range of −100 °C–150 °C, the resonant frequency changed almost linearly with the temperature variation. This work confirms that double electrode transducers are efficient in enhancing the Q value and reducing the return loss of SAW sensors. Furthermore, all experimental results indicate that LiNbO_3_ can be a potential high-performance substitute for precise measurements and large-scale applications in cryogenic temperature environments.

## 2. Materials and Methods

### 2.1. Calculation Principle

The performance of SAW temperature sensors is affected by many factors, such as the properties of the substrate material, sensitive structure, number of reflecting gratings and interdigital transducers (IDTs), the height of the electrodes and the direction of propagation. Hence, in this work, COMSOL Multiphysics 6.0 was utilized to simulate the sensitive structure and analyze the properties of LiNbO_3_ crystal for the sake of improving the design efficiency and optimizing the performance of SAW temperature sensors. Thereinto, 128° YX-cut LiNbO_3_ substrates were described using the Euler angle coordinate system.

The piezoelectric effect is a phenomenon of mutual conversion between mechanical energy and electrical energy, which involves the interaction between electrical quantity and mechanical quantity. Typical of piezoelectric material, LiNbO_3_ substrate is used to excite and detect surface acoustic waves. Based on the piezoelectric constitutive equation, Newton’s second law (mechanical behavior) and Maxwell’s equation (electrical behavior), the mathematical models of SAW sensors are established [15,16].

The piezoelectric coupling constitutive relationship is [17]:

(1) $$\left\{\begin{array}{c} T_{ij}=C_{ijtl}^{E}\cdot S_{kt}-e_{ijk}^{T}\cdot E_{k}\\ D_{i}=e_{itl}\cdot S_{kt}-\varepsilon_{ij}^{s}\cdot E_{j} \end{array}\right.$$

where $T_{ij}$ represents the stress tensor, $C_{ijkl}^{E}$ represents the elastic stiffness tensor of the medium (N/m^2^), $S_{kl}$ represents the strain tensor of the medium, $e_{ijk}^{T}$ represents the piezoelectric tensor (C/m^2^), $E_{k}$ represents the electric field strength (V/m), $D_{i}$ is the electrical displacement (C/m^2^) and $\varepsilon_{ij}^{S}$ represents the dielectric tensor (F/m).

The mechanical properties of the material are decided by Newton’s second law [18].

(2) $$\rho \frac{\partial^{2}u_{i}}{\partial t^{2}}=\sum_{j}\frac{\partial T_{ij}}{\partial x_{j}}$$

where ρ is the density, $u_{i}$ is the global displacement and $x_{j}$ is the global direction.

The electrical properties of the material are decided by Maxwell’s equations. As the surface acoustic wave is far slower than the electromagnetic wave, the quasi-static approximation is established.

(3) $$E_{i}=-\frac{\partial \varphi }{\partial x_{i}}$$

(4) $$\frac{\partial D_{i}}{\partial x_{i}}=0$$

where φ is the electrical potential.

According to Equations (1)–(4), the basic wave equation can be derived mathematically from Newton’s equation and Maxwell’s equation, which couples the electric potential and displacement components together [19]. The specific wave equation is as follows.

(5) $$\sum_{ijk}c_{ijkl}^{E}\frac{\partial^{2}u_{l}}{\partial x_{j}\partial x_{k}}+\sum_{jk}e_{kij}\frac{\partial^{2}\varphi }{\partial x_{j}\partial x_{k}}=\rho \frac{\partial^{2}u_{i}}{\partial t^{2}}$$

(6) $$\sum_{kl}e_{jkl}\frac{\partial^{2}u_{l}}{\partial x_{j}\partial x_{k}}-\sum_{jk}\varepsilon_{jk}^{s}\frac{\partial^{2}\varphi }{\partial x_{j}\partial x_{k}}=0$$

for i,j,k,l = 1, 2 and 3.

In summary, a finite element model of a piezoelectric structure can be established using the above equations.

For a specific SAW temperature sensor, the top priority is its resonant frequency, which is defined in Equation (7), where f is the resonant frequency of the sensor and λ is the period of the IDTs. Consequently, the variation of the resonant frequency is mainly influenced by the phase velocity of the substrate and the period of the IDTs of the SAW temperature sensor. In terms of the phase velocity, it is susceptible to temperature changes, mass loading, mechanical disturbances, humidity changes, etc., as listed in Equation (8). This peculiarity is conducive to the measurement of physical parameters, including temperature, humidity, pressure, strain and acceleration [20].

(7) $$f=\frac{v}{\lambda }$$

(8) $$\frac{\Delta v}{v_{0}}=-k_{m}\frac{\Delta m}{m_{0}}+k_{c}\frac{\Delta c}{c_{0}}+k_{\sigma }\frac{\Delta \sigma }{\sigma_{0}}+k_{\varepsilon }\frac{\Delta \varepsilon }{\varepsilon_{0}}-k_{T}\frac{\Delta T}{T_{0}}+\ldots$$

where v is the phase velocity of the substrate, $k_{i}$ is the sensitivity to each physical parameter, m is the quality, c is the elastic coefficient, σ is film conductivity, ε is the dielectric constant and T is the temperature.

When the outside temperature changes, the material properties of the substrate material vary together on account of the change of thermal stress. Meanwhile, the distance between the IDT and the reflector changes together, leading to the change of the propagation speed of surface acoustic waves. In other words, the change of temperature can be characterized by the shift of the resonant frequency.

The structure of LiNbO_3_ is a hexagonal lattice. During the simulation, when a temperature field is applied, LiNbO_3_ material will undergo thermal change and related physical properties will change. To be specific, the elastic constant, the piezoelectric constant, the dielectric constant and thermal expansion coefficient will be affected by the temperature [21]. The resonant frequency of LiNbO_3_ shows a linear variation with the temperature and the quadratic dependence is very small; hence, in the simulation, only the first-order temperature coefficients of the elastic, piezoelectric and dielectric constants are considered. The material properties are assumed to be linearly dependent on the temperature variation (ΔT). Equations (9)–(11) describe the relationship between physical properties and temperature.

(9) $${\tilde{C}}_{pq}^{E}=C_{pq}^{E}\left(1+TC_{pq}^{E}\times \Delta T\right)$$

(10) $${\tilde{e}}_{ip}=e_{ip}\left(1+TC_{e_{ip}}\times \Delta T\right)$$

(11) $${\tilde{\varepsilon }}_{ij}^{S}=\varepsilon_{ij}^{S}\left(1+TC_{\varepsilon_{ij}}\times \Delta T\right)$$

where $C_{pq}^{E}$, $e_{ip}$ and $\varepsilon_{ij}^{S}$ are the elastic constant, piezoelectric constant and dielectric constant at the reference temperature of $T_{0}$, respectively. $TC_{pq}^{E}$, $TC_{e_{ip}}$ and $TC_{\varepsilon_{ij}}$ are the first-order temperature coefficients of elasticity, piezoelectric stress constant and dielectric constant, respectively. ΔT = T − $T_{0}$, and $T_{0}$ = 25 °C.

### 2.2. Temperature Sensor Simulation

FEM simulation is essential in improving the efficiency of design and fabrication. In this paper, SAW cryogenic temperature sensors were manufactured with 128° YX-cut LiNbO_3_ substrate, and the temperature sensitive simulation analysis was carried out on the 128° YX-cut LiNbO_3_ substrate correspondingly. The LiNbO_3_ material parameters and corresponding temperature coefficients published in literature [22,23] were used.

**Figure 1:**
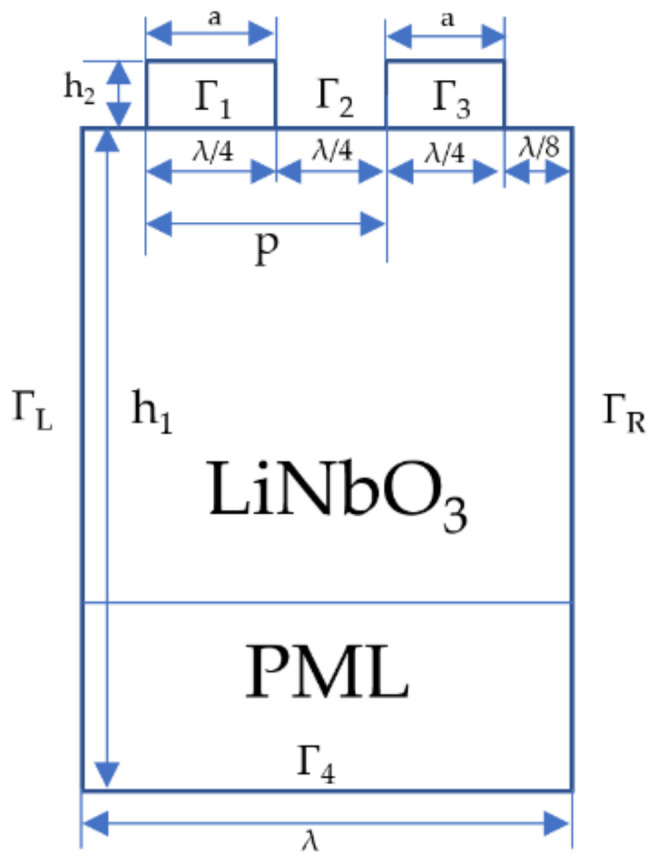
Schematic representation of the single-pair IDT structure in the model.

In this article, COMSOL Multiphysics 6.0 was used for FEM analysis, which contributes to performing piezoelectric coupling analysis and analyzing the response when applied loads change. Thus, the simulation results provide guidance for the design and optimization of SAW temperature sensors. Because the horizontal shear components in SAW devices based on LiNbO_3_ can be neglected, 2D finite element simulation is in high precision. Considering that the electrodes of the IDT are periodically distributed, the simulation model can be established through one cycle to reduce the amount of calculation and decrease the simulation time. The Rayleigh wave and its higher-order mode vibrations are mainly limited to one wavelength (λ) on the surface; hence, the simulation model with a substrate thickness over 5λ is enough. Moreover, a perfect matched layer (PML) of 1λ is set in the bottom of the model to absorb propagating waves, accelerate the attenuation of evanescent waves and match arbitrary lossy media. As a result, a typical 2D unit model was first established, as shown in Figure 1. Thereinto, p refers to the distance between two adjacent metal electrodes, a refers to the width of metal electrode, h_2_ refers to the thickness of the metal electrodes and h_1_ refers to the thickness of LiNbO_3_. The metallization rate (MR = a/p) was designed to be 0.5 to eliminate the influence of the metallization and obtain the maximum reflectivity of the reflector. No losses were considered.

To make the simulation results more accurate and reduce the amount of simulation calculation, the modal analysis of the SAW cryogenic temperature sensors is generally performed using a pair of IDTs with periodic boundary conditions. The detailed electrical and mechanical boundary conditions of the numerical model published in literature [18] were used.

**Figure 2:**
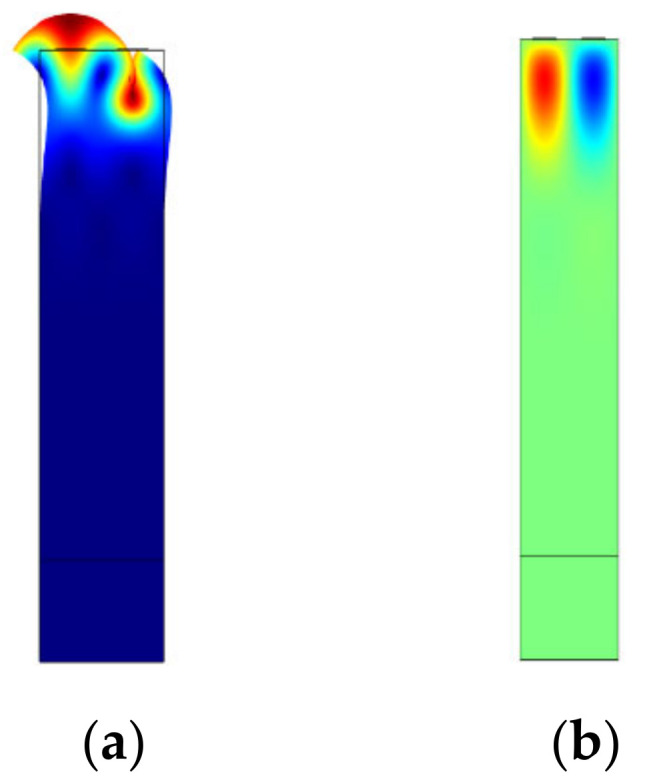
Simulation results of the SAW sensors at Rayleigh mode. (**a**) The mode of vibration at the resonant frequency, (**b**) the corresponding potential diagram.

Since the metallization rate is determined, the propagation characteristics of the SAW sensor have nothing to do with the wavelength. Because the photoetching machine in our laboratory cannot meet the demand of high-precision technique manufacture, the resonant frequency of SAW cryogenic temperature sensors was designed to be 162 MHz; accordingly, the wavelength (λ) of SAW cryogenic temperature sensors with single electrode transducers was set to be 24 um. The initial parameters involved are shown in Table 1. Further optimization was based on this design. In addition, the direction of propagation was determined by changing the Euler angle of the rotating coordinate system in COMSOL Multiphysics 6.0. The simulation results are shown in Figure 2.

**Figure 3:**
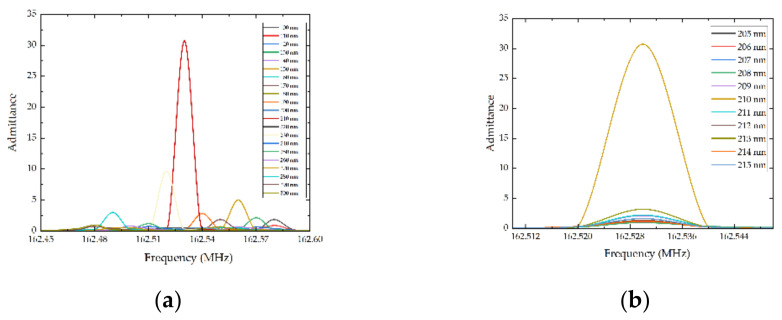
Admittance curves with different thickness of electrodes. (**a**) The thickness of electrodes ranging from 100 nm to 300 nm, (**b**) the thickness of electrodes ranging from 205 nm to 215 nm.

To optimize the performance of the previously designed SAW sensors, the mass loading effect of the IDT was first taken into consideration. In simulation, the mass loading effect was embodied in the influence of admittance caused by the thickness variation of the electrodes. Thus, parameter sweep in COMSOL Multiphysics 6.0 was utilized to analyze the relationship between the thickness of the electrodes and the admittance of SAW sensors. To begin, the thickness of the electrodes (h_2_) varied from 100 nm to 300 nm with a step of 5 nm, and the frequency ranged from 160 MHz to 164 MHz. Figure 3 shows the simulation results of the admittance of different electrode thickness, indicating that the admittance reached a peak when the thickness was 210 nm. Further, the thickness of the electrodes (h_2_) varied from 205 nm to 215 nm with a step of 1 nm. The results are consistent with the results of the first sweep. In conclusion, when the thickness of electrodes reached 210 nm, the admittance of the designed SAW sensor reached a maximum. In other words, the impedance and the loss of the designed SAW sensor reached a minimum. As a result, the performance of the SAW sensors was improved.

The equivalent circuit model can be seen as an equivalent circuit when the SAW device is resonating, which converts crystal substrate and devices parameters to the RLC circuit [24]. The equation of equivalent circuit model is as follows.

(12) $$C_{0}=N_{p}\left(\varepsilon_{r}\varepsilon_{0}+\varepsilon_{0}\right)W$$

where *C*_0_ refers to the static capacitance, *N_p_* refers to the pairs of IDTs, *W* refers to the aperture, $\varepsilon_{0}$ refers to the dielectric constant under vacuum and $\varepsilon_{r}$ refers to the relative dielectric constant of piezoelectric substrate.

To achieve a higher performance of the SAW sensors, the pairs of IDTs (*N_p_*) and the aperture (*W*) should be well considered. Consequently, related simulations were conducted to study the structure parameters of IDTs and optimize the output performance.

**Figure 4:**
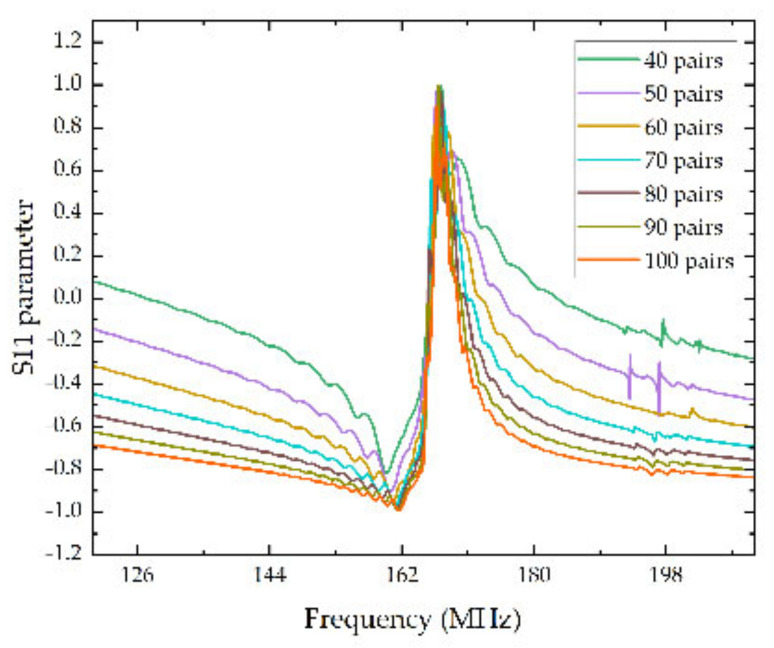
S11 parameters curves of IDTs with different pairs.

Thereinto, the pairs of IDTs were inversely proportional to bandwidth, and with the increasement of the pairs, a smaller bandwidth can be acquired. In other words, the Q value of the SAW sensors was enhanced. In the procedure of simulation, the function of array in COMSOL was utilized to change the pairs of IDTs from 40 to 100 pairs for comparison, and the thickness of the electrodes was set to be 210 nm simultaneously. The simulation results were shown by the S11 parameter, defined as the rate of reflected power and incident power of the device which effectively captures a series of physical process containing the acoustoelectric conversion and spreads the reflection of SAW [25]. The specific simulation results are shown in Figure 4.

As vividly shown in Figure 4, with the pairs of IDTs raising from 40 to 100, the sidelobe of the S11 curves were suppressed gradually, and the harmonic peaks were steeply increased. It can be predicted that with more pairs of IDTs, the device can achieve a better performance. However, more pairs of IDTs also mean a bigger volume. Hence, the pairs of IDTs should be comprehended by the performance of SAW sensors, required size and the difficulty in actual manufacturing technics. Therefore, in this article, the pairs of IDTs were set to be 80.

Similarly, according to the Equation (12), with the increasement of aperture (*W*), the static capacitance (*C*_0_) increased together, which means the coupling was stronger. Once the coupling is enhanced, the insertion loss can be reduced, and the Q value can be improved together. To verify the influence of aperture, the pairs of IDTs were set to be 80 and 100, respectively, and the thickness of the electrodes was set to be 210 nm. The parameter sweep was utilized to scan the aperture from 50λ to 100λ with a step of 10λ.

**Figure 5:**
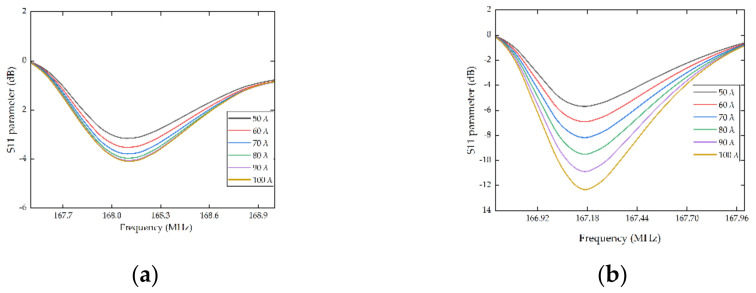
S11 parameter curves of IDTs with different aperture. (**a**) The simulation results with 80 pairs of IDTs, (**b**) the simulation results with 100 pairs of IDTs.

As shown in Figure 5, the insertion loss of the devices was suppressed with the increase of aperture and the harmonic peaks were gradually increased, which is consistent with the phenomenon when changing pairs of IDTs. In brief, whether to add pairs of IDTs or increase aperture will greatly enhance the performance of the SAW resonators. However, an increase in aperture is also accompanied by the increase in device volume. Meanwhile, the diffraction effect of surface acoustic wave will be aggravated. As a result, the aperture was set to be 80λ.

**Figure 6:**
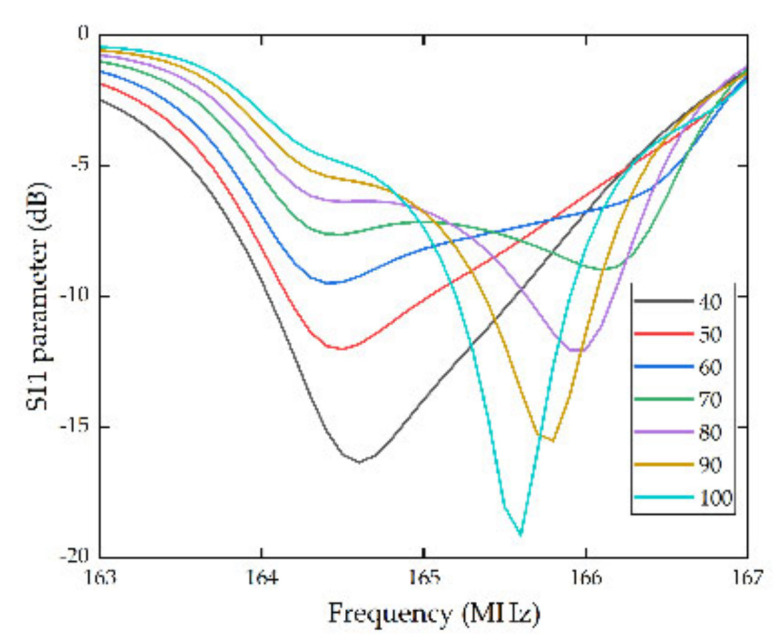
S11 curves of SAW sensors with seven kinds of reflecting grid logarithms.

The reflecting grid logarithm (*N_g_*) was also taken into consideration because it was positively correlated with the reflection coefficient, which has a great influence on the Q value [26]. In the simulation, to reduce the amount of calculation, the pairs of IDTs were set to be 40, and the reflecting grid logarithm changed from 40 to 100 with a step of 10. The simulation results are shown in Figure 6.

The simulation results indicate that when *N_g_* is 100, the S11 curve is the sharpest, which means the SAW sensor with *N_g_* is 100 has the highest Q value. The lowest insertion loss is acquired simultaneously. Consequently, the reflecting grid logarithm is set to be 100.

In summary, a better design of SAW sensors with single electrode transducers, hereafter ‘device A,’ was acquired by FEM simulation, and the specific parameters are shown in Table 2.

To study the influence of the types of the interdigital transducers on the performance of SAW temperature sensors, SAW temperature sensors with double electrode transducers were also designed and fabricated for comparison, which was the first time that double electrode transducers were introduced into SAW temperature sensors. In previous research, double electrode interdigital transducers have not been reported in SAW temperature sensors. Comparative analysis of the SAW resonator using single and double electrode transducers was reported earlier by Stefanescu et al., indicating that single electrode transducers were reflective in nature, generating unwanted spurious signals. Double electrode transducers, on the other hand, were less reflective than single electrode transducers, exhibiting lower insertion losses [27]. An FEM simulation of the single electrode transducers versus double electrode IDTs was also reported by Ralib et al., who acquired a higher quality factor for the double electrode CMOS SAW resonator compared to the single electrode transducers of the CMOS SAW resonator [28]. In brief, compared with single electrode transducers, double electrode transducers have very low reflectivity when short-circuited, which is beneficial to reduce return loss and suppress undesired acoustic reflections.

**Figure 7:**
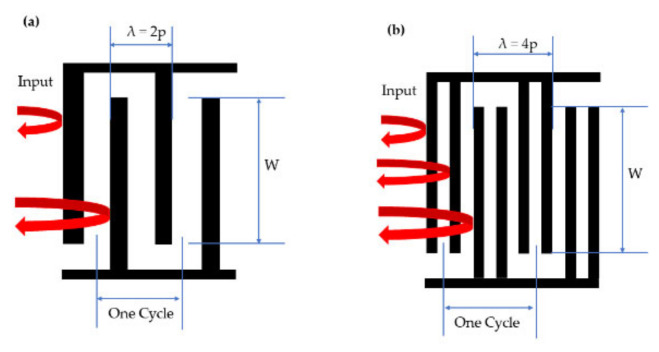
Schematic diagram of internal inflection of surface acoustic wave. (**a**) Single electrode transducers, (**b**) double electrode transducers.

In this article, SAW temperature sensors with double electrode transducers, hereafter ‘device B,’ were simulated and fabricated to study the influence of the types of interdigital transducers on the performance of SAW temperature sensors. The comparison diagram of the double electrode transducers and single electrode transducers is shown in Figure 7. Table 3 shows the parameters of the SAW temperature sensor with double electrode transducers.

**Figure 8:**
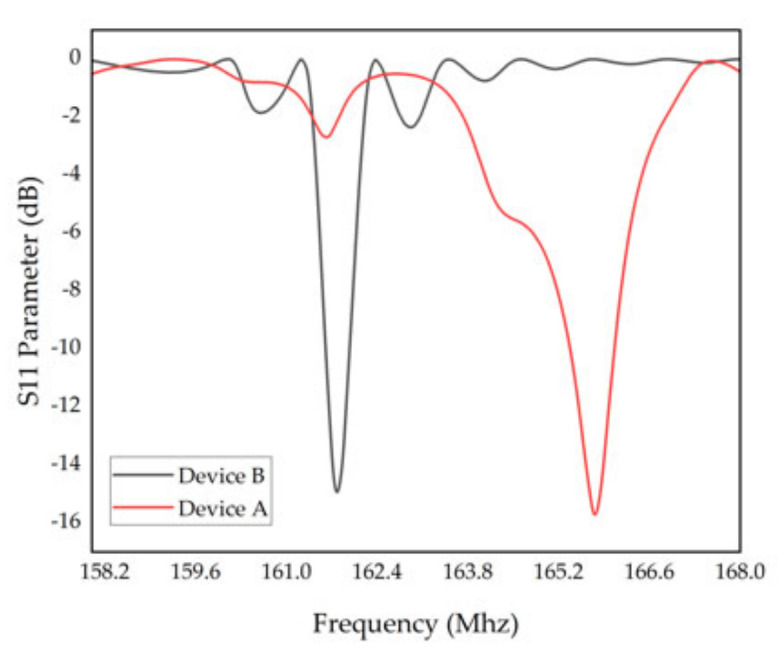
S11 curves of device A and B.

The simulation results of the SAW sensors with single and double electrode transducers are shown in Figure 8. Compared with the SAW sensors with single electrode transducers, the resonant frequency of the SAW sensors with double electrode transducers decreased, which was slightly lower the estimated results. However, the harmonic peak was accentuated by the design of the double electrode transducers. Meanwhile, the harmonic peak was sharper, which means the Q value was enhanced accordingly. Undoubtedly, double electrode transducers are efficient in improving the performance of SAW temperature sensors. Both device A and B were fabricated for further comparison.

Finally, the performance of the SAW sensors in the cryogenic temperature were simulated. To simulate the temperature influence on the SAW sensor, the substrate’s coefficients of thermal expansion (CTE) and the temperature coefficients of material properties, including density, elastic constants, piezoelectric constants and dielectric constants, were taken into consideration in the model. The electrode’s CTE was included in the model as well. The material of the electrodes was Al. The density, Young’s moduli and Poisson’s ratio were taken from the materials library of COMSOL Multiphysics.

**Figure 9:**
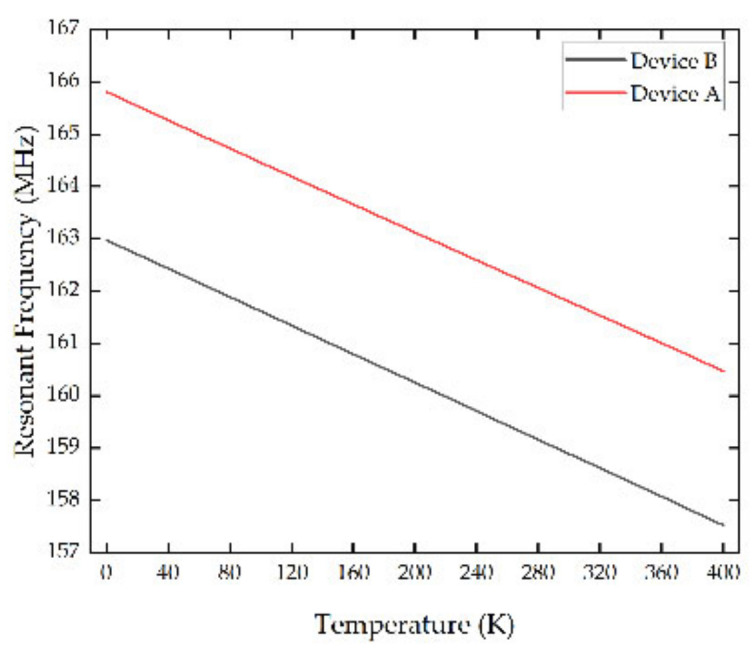
Temperature response of SAW sensors with different electrode transducers.

The performance of device A and B were simulated in contrast. During the simulation, the temperature sweep was set up to simulate the resonant frequency of the devices at different temperatures. The temperature sweep started from 0 K to 400 K with a step of 5 K. The Eigen modes of the devices were calculated with zero driven voltages applied at the IDT electrodes. The resonant frequency was obtained from the symmetry resonance modes of vibration of the device. The simulation results are shown in Figure 9.

As shown in Figure 9, both device A and device B had a good temperature response in cryogenic environment. In addition, the types of electrode transducers had little influence on the temperature sensitivity of the sensors. The TCF was calculated with Equation (13), which was −75.4 ppm/K and −75.7 ppm/K, respectively, for device A and device B.

(13) $$TCF=\frac{\Delta f}{f\cdot \Delta T}$$

where Δf is the frequency difference and ΔT is the corresponding temperature difference. f is the resonant frequency.

### 2.3. Sensor Fabrication

To verify the performance of SAW temperature sensors in low temperatures and evaluate the accuracy of the FEM simulation, device A and device B were fabricated accordingly. The SAW low temperature sensors were fabricated from a 0.5 mm-thick and four-inch 128° YX-cut LiNbO_3_ wafer, which were bought from RDMICO. Pretreatments, including organic cleaning, acid picking and alkali washing, were carried out because the wafer may have been polluted during the transportation. Thereinto, organic matter on the surface of the LiNbO_3_ was removed by organic cleaning. The purpose of acid picking was to clean the metal particle, and the use of the alkali washing was to wipe off the acid solution residue. Before photoresist coating, Hexamethyldisilazane (HMDS) was coated on the wafer by HMDS vacuum oven, which not only changed the surface of wafers from hydrophilic to hydrophobic but also combined its hydrophobic group well with photolithography adhesive bonding. In the process of developing, HMDS enhanced the adhesion between the photoresist and the substrate. Then, the SAW low temperature sensors were manufactured using ultraviolet photolithography (EVG610, EV group). In addition, O_2_ plasma was treated with 60 W radio frequency (RF) power for 6 min (IoN Wave 10, PVA TePla America, Inc., Corona, CA, USA) to increase metal adhesion before deposition. A Ni/Al (20/210 nm) film was prepared as a fork-finger electrode for the SAW sensors using a magnetron sputtering system. Thereinto, Ni was utilized to increase the bonding strength of the Al thin film electrode on the substrate.

**Figure 10:**
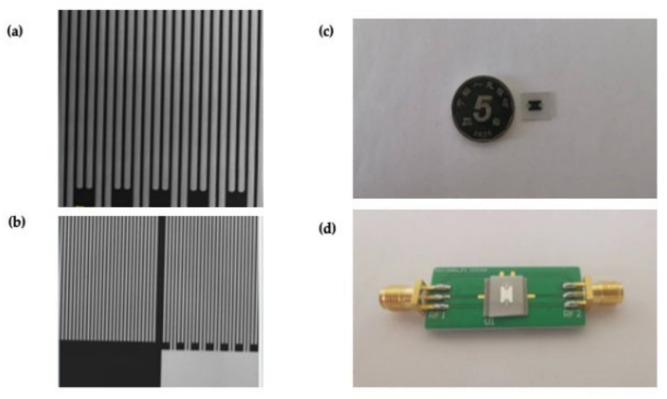
(**a**) The topography of double electrode transducers under confocal microscopy with 50-times magnification and (**b**) 20-times magnification. (**c**) Prepared SAW cryogenic temperature sensors, (**d**) SAW temperature sensors with PCB under test.

Before the low temperature measurement, a specific printed circuit board (PCB) was designed for the auxiliary test. Then, the SAW temperature sensors were scribed from the wafer and connected to the PCB with gold wires by a wire bonder (747677, West bond, Anaheim, CA USA). Then, the SAW temperature sensors could be connected directly to the network analyzer with the coaxial line for measurement. Figure 10 shows the photograph of the prepared sensor and IDTs structure under a microscope.

### 2.4. Cryogenic Temperature Measurement

**Figure 11:**
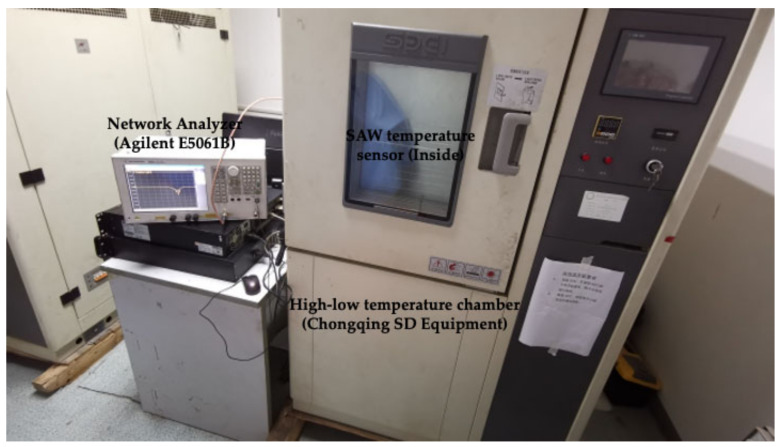
Cryogenic temperature test system.

The temperature dependence of the resonant frequency of the SAW temperature sensor was measured using an RF network analyzer (E5061B, Agilent) and a high-low temperature chamber (Chongqing SD Equipment, China). When the frequency sweep signal sent by the network analyzer was consistent with the resonant frequency of the sensor, it resonated, and the sensor signals were transmitted back to the network analyzer through the PCB and coaxial cable. The operating frequency of the sensors was determined by exacting the curve from the network analyzer. The rate of temperature fall was set at −10 °C/h, with measurements recorded from −100–150 °C in 10 °C intervals to obtain more accurate real-time sensor performance. Figure 11 shows the test scheme for the SAW low temperature sensors.

## 3. Results and Discussion

**Figure 12:**
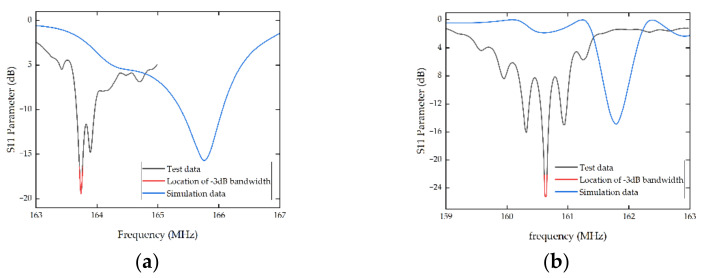
S11 curves of device A and device B at room temperature. (**a**) The comparison results between the simulation data and test data of device A, (**b**) the comparison results between the simulation data and test data of device B.

According to S11 curves in Figure 12, both of which were tested at room temperature, the center frequencies of the fabricated device A and B were 163.74 MHz and 160.625 MHz, respectively, both of which were slightly lower than the desired frequency. One possible reason is the deficiency in fabrication. Compared with the simulation, stray resonance appeared in the practical test, which may have been caused by the influence of the transverse mode. Overall, the FEM simulation has a high predictive accuracy and is efficient for simplified design and performance improvement.

The performance of device A and device B at room temperature was compared by calculating the quality factor (*Q* value), which was calculated based on Equation (14), where $f_{r}$ is the resonant frequency and $\left(f_{2}-f_{1}\right)$ is the −3 dB bandwidth.

(14) $$Q=\frac{f_{r}}{\left(f_{2}-f_{1}\right)}$$

For device A, the −3 dB bandwidth was 0.0554 MHz, and the resonant frequency was 163.74 MHz; consequently, Q was 2955.60, 2.17-times higher than that reported by Geng et al. in 2022 [24]. For device B, the −3 dB bandwidth was 0.0279 Mhz, and the resonant frequency was 160.625 MHz; consequently, Q was 5757.18, 1.95-times higher than SAW sensors with single electrode transducers and 4.23-times higher than that reported by Geng et al. in 2022. More works have been compared and shown in Table 4. Obviously, the optimization of IDT structure parameters is helpful to enhance the performance of the SAW temperature sensors, and the design of double electrode transducers is more efficient in increasing the *Q* value. Simultaneously, from the S11 curves shows, device B has smaller insertion loss than device A, which is consistent with the conclusion that double electrode transducers exhibit lower insertion loss.

**Figure 13:**
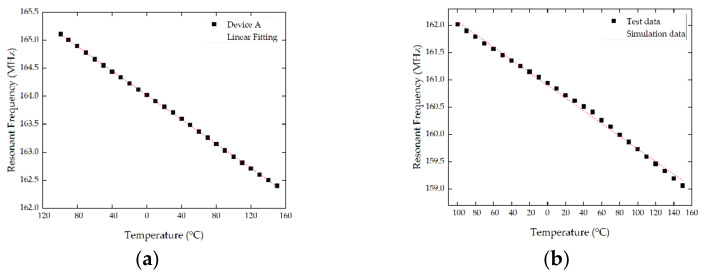
The frequency responsiveness of the SAW sensors during the experiment. (**a**) The frequency responsiveness of device A, and (**b**) the frequency responsiveness of device B.

The performance of SAW sensors in low temperatures was tested by the high-low temperature chamber. The tests were conducted from −100 to 150 °C using the S11 curves recorded for different temperature points by the network analyzer. As the experimental temperature continued to decrease, the resonant frequency of the sensor continued to increase. As is shown in Figure 13, for device A, when the temperature increased from −100 °C to 150 °C, the resonant frequency decreased from 165.21 MHz to 162.3 MHz, and the resonant frequency changed by 2.91 MHz. During the test temperature range (from −100 °C to 150 °C), the average change of the sensor’s resonance frequency was 11.64 kHz/°C, and the TCF was −71.09 ppm/°C. Similarly for device B, the resonant frequency decreased from 162.02 MHz to 159.06 MHz, and the resonant frequency changed by 2.96 MHz. The average change of the sensor’s resonance frequency was 11.84 kHz/°C, and the TCF was −73.7 ppm/°C. The TCF of device A and device B were slightly lower than the simulation data, which may be relevant with the losses and test error. As a whole, the accuracy of simulation is high. All results demonstrate that SAW sensors with double electrode transducers based on 128° YX LiNbO_3_ can be high-performance substitutes for low temperature measurements and are beneficial for mass production due to their low price.

It is regrettable that the temperature range of high-low chamber is limited from −100 °C to 150 °C; as a result, further testing of the frequency responsiveness in lower temperature environment cannot be conducted. However, from the test results, it can be predicted that the SAW temperature sensors based on 128° YX LiNbO_3_ have great potential to work in lower temperature, and even cryogenic, environments. Further tests will be conducted later once appropriate an testing environment is found.

## 4. Conclusions

In this study, high-performance SAW low temperature sensors based on 128° YX LiNbO_3_, of which a higher Q reached up to 5757.18, were obtained by optimizing the structure parameters and introducing double electrode transducers. FEM simulation was conducted by changing the parameters of the height of electrodes, the pairs of IDTs, reflecting grid logarithm and acoustic aperture, demonstrating that optimization of the parameters is helpful to enhance the performance. For the first time, double electrode transducers were introduced into SAW temperature sensors, exhibiting greater a *Q* value than the optimization of structure parameters, which provides a new route when considering enhancing the performance of SAW sensors. Especially when enhancing the *Q* value and reducing the insertion loss, double electrode transducers should be a top priority for outstanding performance. Photolithography and sputtering were utilized to prepare SAW low temperature sensors with single and double electrode transducers, both of which worked on a central frequency of about 160 MHz. The RF network analyzer and high-low temperature chamber were utilized to test their performance in a low temperature environment. The frequency responsiveness curves were almost linear from −100 °C to 150 °C, of which the TCF was −71.9 °C and −73.7 °C, respectively, showing a good temperature response. It is suggested from the experiment results that 128° YX LiNbO_3_ can be high-performance substitutes for precise measurements in low temperature measurements and are beneficial for mass production due to their affordable price. Moreover, SAW temperature sensors based on 128° YX LiNbO_3_ show great potential to work in lower temperature, and even cryogenic, environments. Further tests will be conducted to verify the performance in a cryogenic environment when appropriate testing environment is found.

## Figures and Tables

**Table 1:** The initial parameters of the SAW sensors with single electrode transducers.

Parameters	Initial Values
Resonant frequency (f, MHz)	162
SAW wavelength (λ, um)	24
Width of IDT (a, um)	6
Al Electrode thickness (h_2_, nm)	140
Pitch (p, um)	12
Metal ratio	0.5
Aperture	50λ
The thickness of LiNbO_3_ (h_1_, um)	5λ

**Table 2:** The ultimate design of the SAW sensor with single electrode transducers.

Parameters	Ultimate Values of Device A
Resonant frequency (f, MHz)	162
SAW wavelength (λ, um)	24
Width of IDT (a, um)	6
Al Electrode thickness (h_2_, nm)	210
Pitch (p, um)	12
Metal ratio	0.5
Aperture	80λ
Reflecting grid logarithm (*N_g_*)	100
Pairs of electrodes (*N_p_*)	80

**Table 3:** The parameters of the SAW temperature sensors with double electrode transducers.

Parameters	Values of Device B
Resonant frequency (f, MHz)	160
SAW wavelength (λ, um)	24
Width of IDT (a, um)	3
Al Electrode thickness (h_2_, nm)	210
Pitch (p, um)	12
Metal ratio	0.5
Aperture	80λ
Pairs of electrodes	80
Reflecting grid logarithm (*N_g_*)	100
The thickness of LiNbO_3_ (h_1_, um)	5λ

**Table 4:** Comparison works on the performance of SAW resonators at room temperature.

Piezoelectric Substrate	Frequency (MHz)	*Q* value
LiNbO_3_ [29]	150	1150
128° YX LiNbO_3_ [24]	224.85	1364.5
ZnO/6H-SiC [30]	688	1080
AlN/Al_2_O_3_ [31]	688.75	1082
Sc_0.23_Al_0.77_N/Al_2_O_3_ [32]	1910	659
Device A of this work	163.74	2955.60
Device B of this work	160.625	5757.18
